# Western-style diet consumption impairs maternal insulin sensitivity and glucose metabolism during pregnancy in a Japanese macaque model

**DOI:** 10.1038/s41598-021-92464-w

**Published:** 2021-06-21

**Authors:** Joseph M. Elsakr, Sifang Kathy Zhao, Valerie Ricciardi, Tyler A. Dean, Diana L. Takahashi, Elinor Sullivan, Stephanie R. Wesolowski, Carrie E. McCurdy, Paul Kievit, Jacob E. Friedman, Kjersti M. Aagaard, Digna R. Velez Edwards, Maureen Gannon

**Affiliations:** 1 Department of Molecular Physiology and Biophysics, Vanderbilt University, Nashville, TN USA; 2 Division of Quantitative Sciences, Department of Obstetrics and Gynecology, Vanderbilt University Medical Center, 2525 West End Avenue, Suite 600, Nashville, TN 37203-1738 USA; 3 Division of Diabetes, Endocrinology, and Metabolism, Department of Medicine, Vanderbilt University Medical Center, 2213 Garland Avenue, 7465 MRBIV, Nashville, TN 37232-0475 USA; 4 Division of Cardiometabolic Health, Oregon National Primate Research Center, Beaverton, OR USA; 5 Division of Neuroscience, Oregon National Primate Research Center, Beaverton, OR USA; 6 Department of Pediatrics, University of Colorado School of Medicine, Aurora, CO USA; 7 Department of Human Physiology, University of Oregon, Eugene, OR 97403 USA; 8 Harold Hamm Diabetes Center, University of Oklahoma Health Sciences Center, Oklahoma City, OK USA; 9 Department of Obstetrics and Gynecology, Division of Maternal-Fetal Medicine, and Molecular and Human Genetics, Baylor College of Medicine and Texas Children’s Hospital, Houston, TX 77030 USA; 10 Department of Biomedical Informatics, Department of Obstetrics and Gynecology, Vanderbilt University Medical Center, Nashville, TN USA; 11 Department of Veterans Affairs Tennessee Valley, Nashville, TN USA; 12 Department of Cell and Developmental Biology, Vanderbilt University, Nashville, TN USA

**Keywords:** Developmental biology, Physiology, Diseases, Endocrinology, Health care

## Abstract

The prevalence of maternal obesity is increasing in the United States. Offspring born to women with obesity or poor glycemic control have greater odds of becoming obese and developing metabolic disease later in life. Our group has utilized a macaque model to study the metabolic effects of consumption of a calorically-dense, Western-style diet (WSD; 36.3% fat) during pregnancy. Here, our objective was to characterize the effects of WSD and obesity, alone and together, on maternal glucose tolerance and insulin levels in dams during each pregnancy. Recognizing the collinearity of maternal measures, we adjusted for confounding factors including maternal age and parity. Based on intravenous glucose tolerance tests, dams consuming a WSD showed lower glucose area under the curve during first study pregnancies despite increased body fat percentage and increased insulin area under the curve. However, with (1) prolonged WSD feeding, (2) multiple diet switches, and/or (3) increasing age and parity, WSD was associated with increasingly higher insulin levels during glucose tolerance testing, indicative of insulin resistance. Our results suggest that prolonged or recurrent calorically-dense WSD and/or increased parity, rather than obesity per se, drive excess insulin resistance and metabolic dysfunction. These observations in a highly relevant species are likely of clinical and public health importance given the comparative ease of maternal dietary modifications relative to the low likelihood of successfully reversing obesity in the course of any given pregnancy.

## Introduction

The deleterious effects of maternal obesity and poor glycemic control on the offspring are well documented by clinical and observational studies in human populations^1–7^. Offspring exposed to hyperglycemia with either pre-existing or gestational diabetes in utero have increased incidence of macrosomia and higher skin fold Z-scores at birth, placing them at increased risk for obesity and Type 2 diabetes (T2D) in later childhood and as adults^8,9^. Offspring born to women with poor glycemic control also have higher blood glucose and insulin levels, decreased insulin sensitivity as measured by Homeostatic Model Assessment of Insulin Resistance (HOMA-IR), and higher blood pressure at 9.5 years of age^4^. In addition, these offspring have increased insulin secretion in utero, which correlates with impaired glucose tolerance in childhood independent of obesity in the offspring^6^.

While the increased risk of metabolic disease in the offspring could be due to heritable genetic or epigenetic factors, several studies suggest a major role for a deleterious in utero environment above that of genetic factors^2–4^. For example, offspring born to women with T2D and poor glycemic control during pregnancy are at a significantly increased risk of developing diabetes compared to siblings born from the same mother when she did not have diabetes^2^. Additionally, mothers with T2D transmit risk of obesity, T2D, and impaired glucose tolerance to their offspring at significantly higher rates than fathers with T2D^3^. Importantly, fetuses of obese mothers (in the absence of maternal diabetes) have increased body fat, increased umbilical cord leptin and insulin levels, and increased adiponectin levels that stimulate increased fetal growth^7^. As a result, there is a need to discriminate the impact of maternal obesity from the intake of a calorically dense diet on the in utero environment since this may have different effects on offspring metabolic programming.

To begin to identify the mechanisms by which maternal diet or obesity independently contribute to developmental programming, our group has utilized a non-human primate (NHP) model of maternal overnutrition through Western-Style Diet (WSD) feeding before and during pregnancy. The power of this model is that like humans, not all female macaques develop obesity on WSD, allowing us to dissect how maternal diet, age, and obesity during pregnancy may affect fetal metabolic systems and juvenile disease pathways^10–17^.

Although there is a large body of literature on dietary patterns playing important roles in the development of insulin resistance, very few studies have been done in pregnancy, a unique time in which insulin resistance develops naturally. To discriminate better between the effects of diet versus metabolic status on the maternal phenotype, a thorough analysis of the effects of WSD on maternal metabolism during pregnancy is required. Here, we systematically analyzed the contribution of factors that affect maternal insulin resistance during gestation including time on WSD, diet switching to/from chow to WSD, age, and parity on maternal insulin levels during third trimester glucose tolerance testing. The current analysis allowed us to evaluate the effect of multiple gestations, age, and prolonged unhealthy dietary patterns on maternal insulin resistance, glucose tolerance, adiposity, and leptin levels in a model with clinical relevance to the human condition. Our results suggest that multiple gestations or prolonged unhealthy dietary patterns lead to a maternal metabolic environment that would confer greater risk of obesity and metabolic disease in the offspring.

## Results

**Figure 1:**
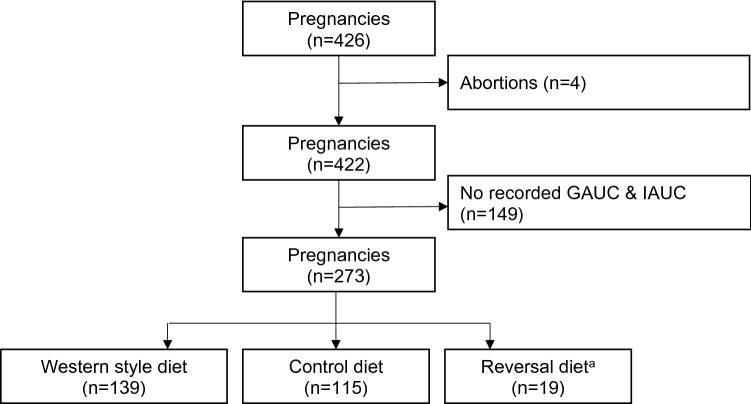
Flow chart of study subject exclusion criteria. Only pregnancies that progressed to either live birth or C-section and had recorded glucose tolerance testing in the third trimester were included in the study. Reversal diet has the same content as the control diet but is used to distinguish animals who were previously exposed to the western style diet (and therefore have 2 prior diet switches).

**Figure 2:**
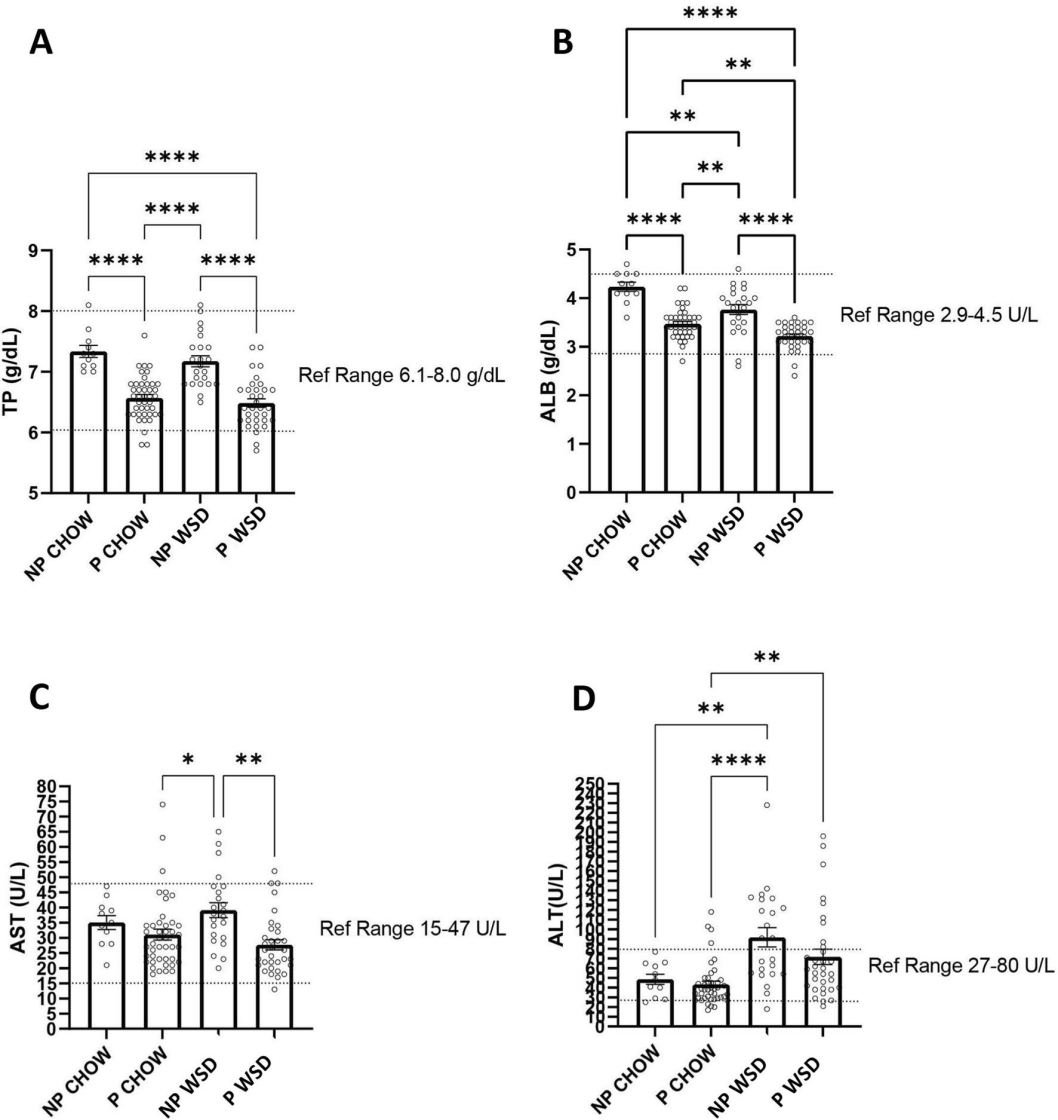
Serum analysis during the third trimester. Blood samples were collected from control (chow) and WSD-fed animals prior to pregnancy (NP) and during the 3rd trimester of pregnancy (P) GTT timepoints, and analyzed for total protein (**A**) and albumin (**B**), as well as the liver enzymes AST (**C**) and ALT (**D**). Total protein was significantly reduced during pregnancy in both diet groups, but no significant impact was observed from the diet itself. Albumin levels were significantly lower in pregnant animals, and were further reduced in animals consuming a WSD, although most animals remained within reference ranges for normal healthy female Japanese macaques. Liver function also remained within normal reference ranges, although WSD feeding did significantly increase ALT levels. Sample sizes were as follows: non-pregnant control (NP CHOW; n = 11), pregnant control (P WSD; n = 43), non-pregnant WSD (NP WSD; n = 23), pregnant WSD (P WSD; n = 33). Groups were analyzed with a one-way ANOVA using a Sidak posthoc test for multiple comparisons (* p < 0.05, **p < 0.01, ***p < 0.001, ****p < 0.0001; Graphpad Prism 9.1).

A total of 273 pregnancies from 95 dams were included in our analysis (Fig. 1). Maternal age ranged from 52 to 202 months at the time of conception. Prior to the start of the current study, 30 (11%) animals were nulliparous. (See “Methods” for full details on maternal characteristics.) The WSD used in the current study contained 18.3% calories from protein compared with 26.8% from protein in the CTR diet. To ascertain whether this difference in dietary protein content resulted in maternal proteinemia, we analyzed serum protein levels (total protein and albumin) (Fig. 2). WSD feeding did not result in a decrease in serum total protein between non-pregnant and pregnant females. Although there is a slight, but significant decrease in circulating albumin levels in WSD fed females (both in the non-pregnant and pregnant states), these values still fall within the reference range for normal. There were also no differences in offspring birth weight (not shown). Taken together, these results suggest that WSD consumption does not lead to maternal protein deficiency in the current study.

We also assessed whether the WSD had effects on maternal liver function as determined by serum measurements of maternal AST (aspartate aminotransferase) and ALT (alanine aminotransferase) in the non-pregnant and pregnant states (Fig. 2). Increased serum levels of AST and ALT indicate impaired liver function. We observed no increase in AST in response to the WSD. ALT was slightly increased above the normal reference range in response to WSD in the non-pregnant state, but this was actually decreased in pregnancy.

Prior to pregnancy, maternal GAUC ranged from 5262 to 15,996 arbitrary units (AU) with a median of 9124 and maternal IAUC ranged from 249 to 26,312 arbitrary units (AU) with a median of 4855. During pregnancy, maternal GAUC ranged from 3936 to 11,336 AU with a median of 6829 and maternal IAUC ranged from 801 to 78,741 AU with a median of 6839. Compared to dams on CTR diet, dams on WSD have higher weight (9 vs. 11 kg), higher body fat percentage (21% vs. 32%), and higher IAUC (3312 vs. 6270 AU) prior to pregnancy (Table 1). Dams on WSD also have higher weight (CTR: 10 kg vs. WSD: 11 kg) and higher leptin levels (CTR: 57 ng/ml vs. WSD: 77 ng/ml) during pregnancy (Table 1). Other characteristics did not differ by diet status (Table 1).

**Table 1:** Distribution of maternal characteristics stratified by diet (n = 254)^a^.

Characteristics	CTR (N = 115)		WSD (N = 139)		P value^c^
	n	% or median (IQR)^b^	n	% or median (IQR)^b^	
Age, months	115	113 (79–148)	139	104 (88–127)	0.40
**Parity**					0.10
0	19	17	11	8	
1	20	17	23	17	
≥ 2	76	66	105	76	
**Pre-pregnancy measures**					
Weight (kg)	115	9.2 (7.8–10.5)	139	10.6 (8.8–12.7)	< **0.01**
Body fat percentage	95	20.9 (11.9–27.9)	77	31.8 (25.6–39.1)	< **0.01**
GAUC	93	9480.0 (8088.5–10,168.5)	122	9006.0 (8097.0–10,294.0)	0.56
IAUC	91	3311.5 (2338.2- 5786.0)	121	6270.1 (4189.3–9388.8)	< **0.01**
**Third-trimester measures**					
Weight (kg)	115	10.0 (8.5–11.4)	138	10.9 (9.3–13.3)	** < 0.01**
Leptin (ng/ml)	90	57.4 (27.7–88.0)	93	77.0 (53.0–161.8)	** < 0.01**
LDL	34	41.0 (34.0–47.0)	32	37.0 (29.0–45.5)	0.26
Triglycerides	34	62.0 (54.0–83.0)	33	72.0 (59.0–108.0)	0.10
Gestational weight gain	115	0.8 (0.2–1.3)	138	0.6 (-0.3–1.5)	0.28

WSD, western-style diet; CTR, control diet; IQR, interquartile range; AU, arbitrary units; GAUC, glucose tolerance test area under the curve; IAUC, insulin area under the curve; LDL, low-density lipoproteins.

^a^Nineteen pregnancies on control diet were excluded for this table due to their diet history (they were placed on control diet after being exposed to WSD in one or more prior pregnancies).

^b^Median and IQR are reported for continuous variables due to small sample size and skewness; percent is reported for parity.

^c^From Wilcoxon rank-sum test for continuous measures; Pearson chi-squared test for parity.

^d^Parity refers to number of live births prior to study pregnancy.

The bold indicates significantly different values from the reference group.

In adjusted models, dams exposed to WSD (n = 138) had similar third-trimester GAUC (coefficient = -342, 95% CI: -723, 38; -5% change), when compared to dams not exposed to WSD (n = 114). Third-trimester IAUC was on average 5108 AU, or 70% higher among dams consuming WSD (n = 133, coefficient = 5108, 95% CI: 2261, 7955; + 70% change), compared to CTR dams (n = 112, Table 2). When we restricted our analysis to first study pregnancies (n = 95), that is the first pregnancy after dams were initially assigned to a diet group, WSD was associated with a decrease in third-trimester GAUC by an average of 811 AU or 12% (coefficient = − 811, 95% CI: − 1230, − 391; − 12% change) and an increase in third-trimester IAUC by 5143 AU or 105% on average (coefficient = 5143, 95% CI: 2430, 7857; + 105% change, Table 3). When restricting our analysis to subsequent pregnancies (n = 159), WSD was associated with an average increase of 5120 AU or 59% in IAUC (coefficient = 5120, 95%CI: 1171, 9068; + 59% change, Table 4).

**Table 2:** Association between WSD and third-trimester GAUC and IAUC (n = 254)^a^.

	Crude				Adjusted^b^			
	n^c^	Coefficient	95% CI	%∆	n^c^	Coefficient	95% CI	%∆
GAUC	252	**− 419.33**	**− 810.51, − 28.15**	**− 6**	252	**− **342.50	**− **722.83, 37.82	**− **5
IAUC	245	**4671.20**	**1578.33, 7764.07**	**61**	245	**5108.18**	**2261.40, 7954.96**	**70**

WSD, western-style diet; CI, confidence interval; GAUC, glucose tolerance test area under the curve; IAUC, insulin area under the curve.

^a^Nineteen pregnancies on control diet were excluded for this table due to their diet history (they were placed on control diet after being exposed to WSD in one or more prior pregnancies).

^b^Adjusting for age and parity.

^c^Sample size differs from 254 due to missing GAUC or IAUC measurements.

The bold indicates significantly different values from the reference group.

**Table 3:** Association between WSD and third-trimester GAUC and IAUC among first study pregnancies (n = 95).

	Crude				Adjusted^a^			
	n^b^	Coefficient	95% CI	%∆	n^b^	Coefficient	95% CI	%∆
GAUC	95	**− 778.84**	**− 1204.24, − 353.44**	**− 11**	95	**− 810.58**	**− 1230.21, − 390.95**	**− 12**
IAUC	89	**4958.28**	**2469.49, 7447.07**	**99**	89	**5143.20**	**2429.63, 7856.77**	**105**

WSD, western-style diet; CI, confidence interval; GAUC, glucose tolerance test area under the curve; IAUC, insulin area under the curve.

^a^Adjusting for age and parity.

^b^Sample size differs from 95 due to missing IAUC measurement.

The bold indicates significantly different values from the reference group.

**Table 4:** Association between WSD and third-trimester GAUC and IAUC among subsequent study pregnancies (n = 159).

	Crude				Adjusted^a^			
	n^b^	Coefficient	95% CI	%∆	n^b^	Coefficient	95% CI	%∆
GAUC	157	**− **202.01	**− **688.33, 283.74	**− **3	157	**− **75.65	**− **553.58, 402.27	**− **1
IAUC	156	4375.77	**− **141.09, 8892.63	48	156	**5119.73**	**1171.21, 9068.25**	**59**

WSD, western-style diet; CI, confidence interval; GAUC, glucose tolerance test area under the curve; IAUC, insulin area under the curve.

^a^Adjusting for age and parity.

^b^Sample size differs from 159 due to missing either GAUC or IAUC measurement.

The bold indicates significantly different values from the reference group.

When stratified by months of exposure to WSD, dams exposed to 13–24 months and 25–36 months of WSD consumption were more likely to have lower GAUC than CTR (13–24 months coefficient = − 920, 95% CI: -1504, − 337; 25–36 months coefficient = − 544, 95% CI: − 1065, -23). In contrast, dams exposed to 60 + months of WSD were more likely to have higher IAUC compared to controls (coefficient = 8166, 95% CI: 3429, 12,903, Table 5). When stratified by the number of diet switches (the number of times a dam changed from one diet group to the other), dams with one diet switch were more likely to have lower GUAC (− 406, 95% CI: − 801, − 12) and higher IAUC (4690, 95% CI: 1940, 7441); dams with three diet switches were also more likely to have higher IAUC (8846, 95% CI: 168, 17,525, Table 6).

**Table 5:** Association of months on WSD with third-trimester GAUC and IAUC (n = 254).

Months on WSD	Crude			Adjusted	
	n^b^	Coefficient	95% CI	Coefficient	95% CI
**GAUC**					
No WSD	114	Reference	Reference	Reference	Reference
4–12	12	**− 705.70**	**− 1351.60, − 59.79**	**− **611.77	**− **1247.96, 24.43
13–24	29	**− 1219.78**	**− 1818.39, − 621.18**	**− 920.24**	**− 1503.56, − 336.92**
25–36	24	**− 707.78**	**− 1252.47, − 163.09**	**− 544.02**	**− 1065.21, − 22.83**
37–48	28	**− **300.58	**− **991.22, 390.06	**− **203.22	**− **889.80, 483.35
60 +	45	252.85	**− **162.93, 668.63	100.48	**− **321.23, 522.20
**IAUC**					
No WSD	112	Reference	Reference	Reference	Reference
4–12	12	2372.13	**− **2228.77, 6973.03	3003.26	**− **1568.38, 7574.89
13–24	24	2110.14	**− **1853.30, 6073.58	3274.34	**− **21.94, 6570.61
25–36	23	1223.20	**− **2199.69, 4646.08	2023.94	**− **1095.14, 5143.03
37–48	28	4329.21	**− **1520.84, 10,179.26	4917.85	**− **744.34, 10,580.05
60 +	46	**8539.33**	**4026.17, 13,052.50**	**8165.80**	**3428.67, 12,902.92**

WSD, western-style diet; CI, confidence interval; GAUC, glucose tolerance test area under the curve; IAUC, insulin area under the curve.

^a^Adjusting for age and parity.

^b^Sample size differs from 254 due to missing GAUC or IAUC measurements.

The bold indicates significantly different values from the reference group.

**Table 6:** Association of prior diet switches with third-trimester GAUC and IAUC (n = 273).

Number of diet switches	Crude			Adjusted^a^	
	n^b^	Coefficient	95% CI	Coefficient	95% CI
**GAUC**					
0	114	Reference	Reference	Reference	Reference
1	126	**− 525.49**	**− 926.50, − 124.47**	**− 406.28**	**− 800.52, − 12.04**
2	19	**− **31.85	**− **688.57, 624.88	**− **156.43	**− **797.33, 484.47
3	12	**695.34**	**15.82, 1374.87**	271.43	**− **327.88, 870.74
**IAUC**					
0	112	Reference	Reference	Reference	Reference
1	121	**4089.33**	**1030.97, 7147.68**	**4690.40**	**1939.70, 7441.09**
2	19	3357.77	**− **497.15, 7212.70	2863.97	**− **1261.17, 6989.10
3	12	**10,538.43**	**3100.59, 17,976.27**	**8846.34**	**167.75, 17,524.93**

CI, confidence interval; GAUC, glucose tolerance test area under the curve; IAUC, insulin area under the curve.

^a^Adjusting for age and parity.

^b^Sample size differs from 273 due to missing GAUC or IAUC measurements.

The bold indicates significantly different values from the reference group.

Because leptin and body fat percentage were both significantly elevated in the WSD group, we performed mediation analysis to determine whether the increased IAUC during pregnancy in the WSD group (Table 2) may be mediated by leptin or increased body fat. When adjusting for leptin levels, WSD was still significantly associated with increased IAUC during pregnancy (data not shown). Thus, leptin did not mitigate the association between WSD and IAUC. When adjusting for whole body percentage fat, WSD was no longer significantly associated with higher IAUC (coefficient = − 587, 95% CI: − 3785, 2610). However, the sample size dropped to 168 due to missing body fat percentage data from 77 pregnancies.

## Discussion

Our aim was to investigate the independent or synergistic effects of diet and maternal obesity on maternal insulin resistance and glucose homeostasis during pregnancy, since the maternal metabolic environment can affect future metabolic health of offspring. Importantly, WSD consumption during pregnancy did not result in impaired liver function or maternal proteinemia, despite lower levels of protein in the WSD. It has not been demonstrated what is the minimal consumption of protein needed for a healthy pregnancy in nonhuman primates. Another standard monkey chow (Lab Diet 5037, 18% calories from protein) is regularly used as the control diet in studies with Old World primates^18,19^. In addition, in studies in rodents, low protein diets during pregnancy usually aim for a 50% reduction in protein content, with diets containing 9–10% calories from protein, while the control diet is about 18–20% protein^20–22^. The WSD used in the current study contained 18% protein, and thus, we consider it unlikely that maternal protein deficiency contributes to the observed phenotype. However, a previous study from our group did show that maternal WSD consumption leads to decreased total protein in breast milk^23^.

When considering the study population as a whole, dams fed a WSD were significantly heavier, had higher body fat percentage, and had higher IAUC before pregnancy (Table 1). During pregnancy, WSD-fed dams also had increased plasma leptin levels. These changes to body composition and hormone levels indicate that, on average, WSD feeding led to negative changes to maternal physiology as expected. Notably, in both diet groups, there was little to no gestational weight gain, with several animals actually losing some weight during pregnancy (Supplementary Table 1). We performed mediation analysis to determine whether the increased IAUC in the WSD group (Table 2) may be mediated by increased body fat percentage. Indeed, after adjusting for body fat percentage the relationship between WSD and IAUC during pregnancy was no longer significant. This is consistent with a previous study in a cohort of dams, which showed that obese, WSD-fed dams had increased IAUC during pregnancy, while lean WSD dams did not^24^.

In the unadjusted analysis, we found that WSD led to increased maternal IAUC during pregnancy, suggesting increased insulin resistance (Table 2). However, WSD also paradoxically led to a decrease in GAUC. It is worth mentioning that IVGTT-derived glucose levels in pregnant females reflect both maternal whole body insulin sensitivity and fetal glucose disposal. The fetus is mainly dependent on maternal glucose, which is mostly dictated by the maternal–fetal glucose gradient^25^. As insulin secretion by the fetal pancreas is sensitive to changes in circulating glucose concentrations^26^, fetal hyperinsulinemia develops under conditions of maternal hyperglycemia. Insulin sensitivity, estimated by HOMA-IR, is actually much greater in neonates than in adults^27^. This increased insulin sensitivity can lead to increased fetal glucose uptake, sometimes referred to as the “glucose steal”, with advancing gestation^28^. This has been clearly demonstrated in the sheep, where fetal hyperinsulinemia results in increased fetal glucose utilization^29^. In our study, fetuses with the highest circulating insulin levels had a greater differential in umbilical cord blood glucose concentrations (vein—artery), supporting glucose steal (S.R. Wesolowski, unpublished observations). However, some WSD-exposed fetuses showed a negative glucose differential, which may reflect net glucose output. Thus, it remains unclear if increased glucose uptake by the fetus would be large enough to contribute to the lower maternal GAUC we observed with WSD exposure.

Our macaque model is a limited resource shared by a consortium of investigators, dams therefore go through multiple pregnancies at different ages, and occasionally switch diet groups depending on the needs of the consortium. This can lead to challenges in causal analyses due to variation in age and parity between the two diet groups. For example, the CTR group on average had higher parity, which almost reached statistical significance (p = 0.10, Table 1). To account for these differences, we performed the current analyses adjusting for age and parity. Indeed, in the adjusted analysis WSD was no longer associated with decreased GAUC, while the increased IAUC was maintained (Table 2). This suggests that WSD indeed has a significant and profound detrimental metabolic effect on dams during pregnancy.

To minimize the effect of parity, we also analyzed GAUC and IAUC during first versus subsequent study pregnancies (Tables 3 and 4). Since first study pregnancies are not necessarily the dam’s first lifetime pregnancy (i.e., they are not necessarily primigravidae), age and parity were also accounted for in adjusted models. In first study pregnancies, GAUC was lower in the WSD group for both crude and adjusted models. It is unclear whether this is simply an artifact of this sub-population of dams, or a true physiological response to WSD. As pointed out above, early in the course of WSD feeding, increased fetal glucose uptake may account for these findings. In humans, newborns of obese women can have hyperinsulinemic hypoglycemia, suggesting beta cell overcompensation due to increased fetal glucose disposal in response to the in utero hyperglycemic, hyperlipidemic environment^30^.

In subsequent pregnancies in the same animals (Table 4), we found that WSD was associated with higher IAUC and unchanged GAUC. We observed a similar phenomenon when stratifying WSD-fed dams by the number of months on WSD (Table 5). When dams had been fed WSD for 4–36 months, GAUC was significantly reduced in adjusted models. With longer duration of WSD, GAUC was similar to CTR animals. When dams were exposed to WSD for 60+ months, IAUC was significantly increased with no change in GAUC, further suggesting that prolonged WSD feeding leads to insulin resistance during pregnancy. We sought to investigate whether diet switching could affect GAUC and IAUC. When CTR animals were first switched to WSD, GAUC was significantly reduced while IAUC was increased (Table 6). However, with subsequent diet switches GAUC increased to the point of being non-significantly higher than in CTR animals. Interestingly, IAUC was nearly twice as high relative to CTR animals with the second switch to WSD. In mice, diet-induced weight cycling leads to decreased glucose tolerance and insulin sensitivity compared to high-fat diet-fed mice that do not cycle^31^. The long-term metabolic effects of cyclical dieting and weight cycling in humans remains inconclusive^32,33^; however, there is evidence showing that inter-pregnancy weight gain is deleterious to metabolic health of offspring in subsequent pregnancies^34^. It is unclear whether the increased IAUC in our cohort is due to direct effects of diet switching, or simply affected by increasing duration of WSD in the sub-group with three diet switches. Though further studies are needed, the current data suggest a stepwise deterioration in glucose tolerance and insulin sensitivity with multiple bouts of WSD feeding.

In total, our findings demonstrate a persistent and notable impact of WSD feeding during pregnancy on maternal insulin resistance and metabolic physiology. This can be mediated by parity, which itself cannot be dissociated from maternal age. Following prolonged stressors including multiple years on WSD, multiple bouts of WSD feeding, and multiple previous pregnancies, we observe that dams show increased IAUC and normal or worsening GAUC, as might be expected due to worsening maternal metabolic health. Importantly, work from our group shows that some offspring phenotypes are dependent on the metabolic status of the dam. For example, livers of fetuses exposed to WSD had evidence of hepatic oxidative stress only when mothers became obese^16^. Fetuses of obese, WSD-fed dams also had increased expression of gluconeogenic enzymes and transcription factors in the liver^16^. When dams were switched from WSD to a healthy diet before pregnancy, fetal triglyceride levels and hepatic steatosis improved and changes in gluconeogenic gene expression and metabolites were partially, but not completely, reversed^14,16^. These improvements in fetal outcomes occurred in the absence of maternal weight loss; however, maternal insulin sensitivity was improved in dams experiencing diet switching^16^. Thus, some effects of maternal WSD on the offspring appear to be a direct effect of the diet, while others are likely consequences of maternal metabolic status and its influence on the in utero environment in which the offspring develops. Overall, these findings strongly suggest that the effects of chronic vs. short-term maternal WSD matter greatly to maternal–fetal physiology, and that multiple pregnancies may be an added risk factor when considering the impact of fetal developmental programming. This has important implications for human pregnancies. Women who are older or have had multiple prior pregnancies may be more likely to have a less healthy in utero environment during gestation if they consume a diet high in fat, putting the fetus at significantly increased risk of metabolic disease later in life.

## Methods

### Study population

This study was carried out in compliance with the ARRIVE guidelines. Adult female Japanese macaques (*M. fuscata*) were housed in indoor/outdoor cages with social environments comprised of several females to a male to facilitate optimal breeding. Females were sedated in the first or second trimester of pregnancy for fetal dating by ultrasound. Females were also sedated for glucose tolerance tests during the third trimester of pregnancy (range: gestational day (GD) 92 to GD146; average: GD122 + /− 4.4 days) for measures of glucose tolerance. Pregnant females gave birth naturally in their social groups or underwent C-sections after third trimester GTT data was collected. A total of 426 pregnancies from 95 dams were observed. After excluding early abortions (n = 4) and pregnancies with no recorded third trimester GAUC or IAUC (n = 164), we have a total of 273 pregnancies for our analysis (Fig. 1). Ninety-five of these were first study pregnancies (but not necessarily first lifetime pregnancies), and 178 were subsequent study pregnancies. Of the 95 animals included, 30 were nulliparous prior to the start of the study.

All animal procedures were conducted in accordance with the guidelines of the Institutional Animal Care and Use Committee (IACUC) of the Oregon National Primate Research Center (ONPRC) and Oregon Health and Sciences University. All procedures were approved by the ONPRC IACUC. The ONPRC abides by the Animal Welfare Act and Regulations enforced by the USDA and the Public Health Service Policy on Humane Care and Use of Laboratory Animals in accordance with the Guide for the Care and Use of Laboratory Animals published by the National Institutes of Health.

### Exposure

Animals were placed on either a control (CTR: Fiber Balanced Diet 5000; Purina Mills) or Western-style diet (WSD: TAD Diet no. 5L0P, Test Diet, Purina Mills). The CTR diet is made up of 15% of calories from fat (26.8% from protein), whereas the WSD has 36.3% of calories from fat (18.3% from protein). Animals in the WSD group also received calorically dense treats (consisting of ground WSD pellets (7%), banana (10%), peanut butter (26%), cornstarch (34%), and honey (23%)) once per day. Prior to study initiation, all animals were fed CTR diet. At the start of the study, some dams were switched to WSD (one diet switch). A small subset of these dams was switched back to CTR (two diet switches), and a subset of those dams were again placed back on WSD diet (three diet switches). Whether animals remained on the starting diet or were switched to the alternate diet was determined by the needs of the study.

### Outcomes

Intravenous glucose tolerance tests (ivGTT) were performed on pregnant adult females during the early third trimester average 123 + /− 5 days (total gestational is approximately 175 days) for a measurement of glucose tolerance and insulin sensitivity as previously described^16^. Briefly, animals were fasted overnight and sedated with Telazol (3–8 mg/kg IM Tiletamine HCl/Zolazepam HCl, Fort Dodge Animal Health, Fort Dodge, Iowa, USA). If needed, additional anesthesia was accomplished with Ketamine hydrochloride (3–10 mg/kg IM). Once sedated, animals received an IV glucose bolus (50% dextrose solution) at a dose of 0.6 g/kg via the saphenous vein. Baseline blood samples were obtained prior to the infusion and at 1, 3, 5, 10, 20, 40, and 60 min after infusion. Glucose was measured immediately using OneTouch Ultra Blood Glucose Monitor (LifeScan), and the remainder of the blood was kept in heparinized tubes on ice for insulin measurement. After centrifugation, samples were stored at -80 until assayed. Insulin measurements were performed by the Endocrine Technologies Support Core (ETSC) at the ONPRC using a chemiluminescence-based automatic clinical platform (Roche Diagnostics Cobas e411, Indianapolis, IN, USA).

Glucose Area Under the Curve (GAUC) was calculated by integrating the curve of glucose values during ivGTT, with a baseline of 0. Insulin Area Under the Curve (IAUC) was similarly calculated using plasma insulin levels collected during ivGTT.

### Serum protein and liver enzymes

Serum chemistry analysis for albumin, total protein, AST and ALT was performed on a selected number of pregnancies at the third trimester throughout the study using an ABX Pentra 400 serum analyzer (Horiba Medical, CA) via the ONPRC clinical pathology laboratory. Reference ranges provided in the figures were obtained from a total of 43 healthy female Japanese macaques ranging in age from 4 to 17 years of age.

### Covariates

Maternal age was calculated as the maternal age at third-trimester GTT. At the time of conception, maternal age ranged from 52 to 202 months with a median of 109 months. All animals were raised on CTR diet, and a subset were switched to WSD for the study. Age at initiation of WSD ranged from 41 to 191 months with a median of 77 months. Parity was defined as number of previous pregnancies prior to study pregnancy. Parity ranged from 0 to 10 with a median of 2.

Pre-pregnancy measures include weight (kg), body fat percentage obtained from a DEXA scan, GAUC, and IAUC. Third-trimester measures include weight (kg), serum leptin (ng/ml), low-density lipoproteins (LDL), and triglycerides. Gestational weight gain was calculated as the difference in weight between pre-pregnancy and third-trimester measurements for the study pregnancy. Months on WSD was calculated using diet initiation date and third-trimester GTT date, accounting for diet switch patterns. The number of diet switches is zero for CTR animals and ranges from one to three for WSD animals.

### Statistical analysis

Maternal characteristics were compared by diet intake and feeding using Pearson chi-squared test for categorical measures and the Wilcoxon rank-sum test for continuous measures (Table 1). Nonparametric tests were used due to small sample size and skewness. Linear regression was used to calculate beta coefficients and 95% confidence intervals (CIs) to determine whether WSD was associated with impaired glucose tolerance (Table 2). The beta coefficient is the average change in GAUC/IAUC comparing WSD to CTR dams. Thus, a negative coefficient represents a lower average GAUC or IAUC in the WSD group, which is associated with better glucose tolerance. Age and parity were selected as a priori confounders based on the literature. In our primary analysis, we did not adjust for a priori mediators, including pre-pregnancy weight, pre-pregnancy GTT, gestational weight gain, body fat percentage, serum triglycerides, LDL, or leptin levels, since they may mediate the effect of WSD on third-trimester GAUC and IAUC. The sandwich estimate of variance was used to obtain robust variance that accounts for correlation among pregnancies from the same mother^35^. Since the number of pregnancies on WSD may influence GAUC and IAUC, we repeated our analysis among first study pregnancies (n = 95) after diet initiation (Table 3). We also repeated the analysis among subsequent study pregnancies (n = 159, Table 4).

In secondary analyses, we examined the relationship between months on WSD and third-trimester measures since time on diet was not captured in the primary analysis (Table 5). Similarly, we examined the association between diet switching and third-trimester measures (Table 6). In addition, we tested if a priori mediators are significantly associated with both diet and glucose tolerance and if the effect of diet was mitigated when adjusting for the mediator.

All statistical analyses were performed at a 2-sided significance level of 0.05 using STATA 14.2 (StataCorp, Texas, USA).

## Supplementary Information

Supplementary Information 1.

## Data Availability

All data generated or analyzed during this study are included in this published article (and its Supplementary Information files).
